# IMPROVING COLLEGE STUDENTS’ ATTITUDES TOWARD AGING POPULATIONS: USING CBT-BASED GERONTOLOGICAL PEDAGOGY

**DOI:** 10.1093/geroni/igz038.2665

**Published:** 2019-11-08

**Authors:** Qiwei Li, Heath Harllee, Becky P Knight

**Affiliations:** University of North Texas, Denton, Texas, United States

## Abstract

The U.S. is facing a shortage of aging-related professionals. Lack of positive attitudes among undergraduate students towards the older population may restrict them from participating as aging professionals. However, research majorly focused on the attitudes among students already in the medical training fields instead of improvement of the attitudes among undergraduate students who potentially will devote themselves to be aging professionals. We seek to enhance attitudes toward the aging population through gerontological coursework at the undergraduate level. The introductory course is based on Cognitive Behavioral theory (CBT) which emphasizes to unlearn false concepts. Therefore, the class exposes students to positive aging images in contemporary films and literature demonstrating concepts such as successful aging, retirement, gender issues, and aging as minorities, etc. Students review their perceptions of the aging population at the beginning and end of the semester, presenting five words that best describe the aging images. The variances of occurrence of negative to positive descriptions are analyzed as an evaluation of the class. The class also stimulates civic responsibilities of the students toward the aging population with concepts such as generational equities by conducting team-based discussions. The results report increased positive words and improved attitudes on in-class team projects and post-class evaluations. The implication of the course outcomes is that discussing and exposing the positive images, and justifying typical aging-related behaviors significantly improves students’ attitudes toward the aging population, and may encourage undergraduate students to select an aging profession. Qualitative evaluation of the course will be conducted in future semesters.

